# Association between C1019T polymorphism of the connexin37 gene and coronary heart disease in patients with in-stent restenosis

**DOI:** 10.3892/etm.2012.852

**Published:** 2012-12-05

**Authors:** SU-XIA GUO, ZHEN-YU YANG, RU-XING WANG, YING YANG, HUA-MING CAO, TAO ZHANG

**Affiliations:** Department of Cardiology, The Affiliated People’s Hospital of Nanjing Medical University in Wuxi and People’s Hospital of Wuxi City, Jiangsu, Wuxi 214023, P.R. China

**Keywords:** coronary heart disease, in-stent restenosis, connexin37, gene polymorphism

## Abstract

Studies have shown that a C1019T polymorphism of the gene encoding the gap junction protein connexin37 is associated with coronary artery disease (CAD). The aim of the present study was to explore the association between the C1019T polymorphism in the connexin37 gene and CAD patients with in-stent restenosis (ISR). A total of 532 patients who had undergone coronary stenting and coronary angiography at least three months after the procedure were divided according to a clinical diagnosis standard into two groups which were ISR (n=67) and no in-stent restenosis (NISR; n=465) groups. A further 501 healthy individuals were controls. The subjects were genotyped by DNA sequencing. The results demonstrated the following: i) connexin37 gene 1019 sites in the population were distributed by polymorphism into three genetic types (CC, TC and TT types). The distribution frequency of the healthy control, ISR and NISR groups conformed to the Hardy-Weinberg genetic balance rule; ii) in comparison with the healthy controls, the frequency of the connexin37 C allele was higher in the CAD patients (57.05% vs. 41.32%; OR, 1.89; 95% CI, 1.58–2.25; P<0.01). The frequency of the C carriers (CC+TC) was 65.47% in the healthy controls, vs. 79.32% in CAD patients (P<0.01). The CAD risk was significantly increased in the carriers of the C allele (CC+TC) compared with TT homozygotes (OR, 2.03; 95% CI, 1.53–2.80; P<0.01). Stratified analysis demonstrated that a significant difference existed in the frequency of C carriers between the male CAD patients and healthy controls (79.63% vs. 72.45%; OR, 1.48; 95% CI, 1.06–2.09, P=0.02), as well as in the female CAD patients (78.00% vs. 51.50%; OR, 3.34; 95% CI, 1.90–5.86; P<0.01). In the female and male CAD patients, the frequency of the connexin37 C allele was higher than in the healthy controls (male: χ^2^=12.67, P<0.01; female: χ^2^=50.20, P<0.01); iii) compared with the NISR group, the frequencies of the connexin37 C allele and C carriers (CC+TC) were significantly higher in the ISR group (frequency of C allele: 72.39% vs. 54.84%; P<0.01; frequency of C carriers: 89.55% vs. 77.85%; P=0.03). Compared with TT homozygotes, the restenosis risk was significantly increased in the carriers of the C allele (CC+TC; OR, 2.44; 95% CI, 1.08–5.50). Subsequent stratified analysis revealed that the frequency of the C allele was significantly higher in the male ISR group than in the male NISR group (78.57% vs. 52.66%; OR, 3.30; 95% CI, 2.05–5.29; P<0.01). The restenosis risk was ∼four-fold higher in the C carriers (CC+TC) than in the TT homozygotes (OR, 3.74; 95% CI, 1.32–10.64). However in the female population, there was no difference in the ISR risk between the carriers of the C allele (CC+TC) and the TT homozygotes (P=0.70). In summary, the C allele of the connexin37 gene is not only is associated with the susceptibility to CAD, but also associated with restenosis following coronary stenting in the population studied herein, particularly the male population.

## Introduction

Percutaneous coronary intervention (PCI) is well-established treatment strategy for coronary artery disease (CAD), particularly following the introduction of drug-eluting stents (1,2). Despite technological and pharmacological advances, the main limitation of PCI is in-stent restenosis (ISR), which is due to neointimal hyperplasia following the arterial wall injury induced by balloon inflation and stent placement (3,4). Smooth muscle cell (SMC) migration and proliferation is considered be key to intima repair. In this reparative process, genetic factors are involved as well as conventional clinical and procedural parameters (5,6). Consequently, additional genetic tests to identify patients at a high risk of restenosis may lead to improved risk stratification and eventually individual patient-tailored therapy.

Connexins are members of a family of proteins encoded by at least 20 different mammalian genes expressed in a wide variety of tissues (7–9). They form transmembrane channels called gap junctions which connect neighboring cells and allow the passive diffusion of small molecules (10). Of the connexin family, connexin37 is the most highly expressed in the vascular endothelium and is involved in the growth and regeneration of endothelial cells after injury and ageing, suggesting that changes in connexin37 are associated with diseases following intima injury, including CAD and ISR (11–13).

Boerma *et al* identified a link between a single nucleotide polymorphism (SNP) in the human connexin37 gene and the thickening of the carotid in a Swedish male population (14), with the C allele being over-represented in individuals with atherosclerotic plaques. The C allele of this SNP has also been associated with CAD in Taiwan, northern China and Switzerland (15–17). Subsequently in two studies performed in Japanese and Caucasian populations, the T SNP has been shown to be a risk factor for acute myocardial infarction (AMI), particularly in high-risk male individuals (18,19).

Although it is not clear which allele is the more closely associated, the majority of gene polymorphism-association studies have detected that the C1019T SNP in the human connexin37 gene is associated with CAD and myocardial infarction (MI) in various populations. However, whether such a polymorphism may be used as a prognostic marker of ISR following percutaneous coronary intervention, is not known. The present study was designed to investigate whether the connexin37 C1019T polymorphism is associated with restenosis following coronary stenting in the Han population of Wuxi City in China.

## Materials and methods

### Population

This was a single-center, prospective observational study. A total of 532 Han Chinese patients who had undergone successful coronary stenting at the Cardiac Unit of Nanjing Medical University Hospital in Wuxi between 2009 and 2011 and returned for follow-up coronary angiography at least three months later (median, eight months) were selected. In particular, if clinical symptoms appeared (such as chest pain and abnormality in electrocardiograms or cardiac enzymes), coronary angiography was performed at any time. All patients regularly received anti-platelet drugs (aspirin 100 mg/day, clopidogrel 75 mg/day), statins (types and doses determined by doctors) and other necessary drugs, such as angiotensin-converting enzyme inhibitor, β-blockers and nitrates, after the procedure. A further 501 healthy individuals from the medical examination center of the same hospital were the controls. No patients or controls were blood relatives. All participants gave written consent and the ethics committee of People’s Hospital of Wuxi City approved the study.

### Collection of clinical and epidemiological data

Questionnaires were filled out to record basic information for each case (such as age, gender and nationality), risk factors for restenosis (hypertension, diabetes mellitus, hyperlipidemia and smoking/drinking history) and family history of cerebrocardiac vessel disease. Information concerning disease onset, date of patient examinations during hospitalization and procedure-associated information were also collected.

### Quantitative coronary analysis (QCA)

QCA was performed as described previously on images obtained before and immediately after stent placement and at follow-up using a computerized quantitative analysis system. The angiography was performed with at least two projections following an intracoronary injection of isosorbide dinitrate (0.2 mg). The tip of a 6F or 7F catheter filled with contrast medium was used for calibration. Restenosis was defined as a ≥50% diameter reduction in the dilated segment.

### Genotyping

DNA was purified from samples of whole white blood cells, which had been stored frozen at −20°C. Purification was performed using a DNA Purification kit (Promega Inc., Madison, WI, USA) according to the manufacturer’s instructions. The polymerase chain reaction (PCR) was conducted with the upstream primer, 5′-CCTCCTCAGACCCTTACACGG-3′ and downstream primer, 5′-CATCCCAGGCAGCCAGACT-3′ (designed and produced by Ying-Jun Biological Company, Shanghai, China). A 20-*μ*l reaction volume was used for PCR containing 10 *μ*l 2X mix (including Mg^+^, dNTPs and Taq DNA polymerase), 1.0 *μ*l upstream primer (10 pmol), 1.0 *μ*l downstream primer (10 pmol), 2 *μ*l genomic DNA template (up to 4.0 *μ*l, according to the concentration) and 6 *μ*l ddH_2_O. The reaction began with denaturation at 94°C for 5 min, followed by 35 cycles of denaturation at 95°C (30 sec), annealing at 60°C (30 sec), extension at 72°C (30 sec) and a final extension at 72°C (7 min). Genotype analysis was performed using DNA sequencing, which was completed by Ying-Jun Biological Company.

### Statistical analysis

Allele frequencies were calculated by allele counting. Analyses for possible deviations of the genotype distribution from that expected for a population in Hardy-Weinberg equilibrium were performed with the χ^2^ test. Data are presented as the mean ± SD or number (proportion, %). Continuous variables with a Gaussian distribution, as determined by the Shapiro-Wilk test, were compared by one-way analysis of variance (ANOVA) or t-tests. Categorical values were compared by the χ^2^ test. Continuous variables with a non-Gaussian distribution were compared by the Mann-Whitney U test. Analyses were performed using the SPSS statistical software (Version 13.0, SPSS Inc, Chicago, IL, USA). P<0.05 was considered to indicate a statistically significant difference.

## Results

### Baseline characteristics

A total of 501 healthy individuals formed the control group and 532 patients were enrolled, 67 of whom developed ISR. The distribution of clinical and procedure-associated factors, including age, gender, body mass index (BMI) and hypertension, were not observed to be significantly different between the ISR and no in-stent restenosis (NISR) groups. Stent length, stent diameter and release pressure exhibited no differences between the ISR and NISR groups (Table I).

### Connexin37 C1019T genotyping and association analysis

**Figure 1.**
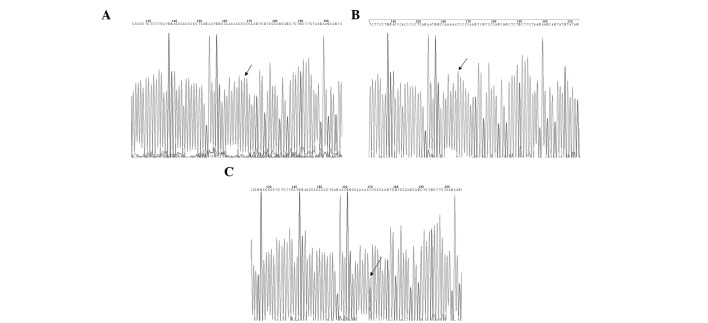
Gene sequencing diagram. (A) CC type. (B) TT type. (C) TC type.

The C1019T polymorphism of the connexin37 gene was detected in the whole population (Fig. 1). The genotype frequencies of CC, TC and TT in the connexin37 C1019T polymorphism were 17.16, 48.30 and 34.53% in the healthy control group; 55.22, 34.33 and 10.45% in ISR group; and 31.83, 46.02 and 22.15% in the NISR groups, respectively. They all obeyed the Hardy-Weinberg law.

Compared with the healthy controls, the frequency of the connexin37 C allele was higher in the CAD patients (57.05% vs. 41.32%; OR, 1.89; 95% CI, 1.58–2.25; P<0.01). The frequency of C carriers (CC+TC) was 79.32% in the CAD patients and 65.47% in the healthy controls (P<0.01). The CAD risk was significantly increased in the carriers of the C allele (CC+TC) compared with TT homozygotes (OR, 2.03; 95% CI, 1.53–2.80). Subsequent stratified analysis demonstrated that a significant difference existed in the frequency of C carriers between the male CAD patients and healthy controls (79.63% vs. 72.45%; OR, 1.48; 95% CI =1.06–2.09, P=0.02), as well as in the female CAD patients (78.00%vs. 51.50%; OR, 3.34; 95% CI, 1.90–5.86; P<0.01). In female and male CAD patients, the frequency of the connexin37 C allele was higher than in the healthy controls (male: χ^2^=12.67, P < 0.01; female: χ^2^=50.20, P<0.01; (Tables II, IV and V).

Compared with the NISR group, the frequencies of the connexin37 C allele and C carriers (CC+TC) were significantly higher in the ISR group (frequency of C allele, 72.39% vs. 54.84%, P<0.01; frequency of C carriers: 89.55% vs. 77.85%, P=0.03). Compared with TT homozygotes, the restenosis risk was significantly increased in the C carriers (CC+TC; OR, 2.44; 95% CI, 1.08–5.50). Subsequent stratified analysis revealed that the frequency of the C allele was significantly higher in the male ISR group than in the male NISR group (78.57% vs. 52.66%; OR, 3.30; 95% CI, 2.05–5.29; P<0.01). The restenosis risk was ∼four-fold higher in the C carriers (CC+TC) than in the TT homozygotes (OR, 3.74; 95% CI, 1.32–10.64). However, in the female population, no difference was identified in the ISR risk between the C carriers (CC+TC) and the TT homozygotes (P=0.70; Tables VI and VII). By binary logistic adjustment for age, gender, BMI, smoking, alcohol, consumption, hypertension, diabetes, hyperlipidemia, stent length and stent diameter and balloon pressure, which are risk factors of stents restenosis, the stent restenosis risk for C carriers was calculated to be 1.78-fold higher that of the patients carrying the T allele (95%CI, 1.23–3.14; P<0.01; Tables III, VI and VII).

## Discussion

In the present study, the frequency of the C allele at base-pair 1019 of the connexin37 gene in patients with CAD was observed to be significantly higher than that of the healthy controls, which was consistent with the study by Han *et al* of a northern Han population in China (16). Furthermore, the frequencies of connexin37 C allele and C carriers (CC+TC) were observed to be significantly higher in the ISR group compared with the NISR group (frequency of C allele: 72.39% vs. 54.84%, P<0.01; frequency of C carriers: 89.55% vs. 77.85%, P=0.03). The restenosis risk was ∼three-fold higher in the carriers of the C allele (CC+TC) than in the TT homozygotes (95%CI, 1.32–10.64) in the male population. The C1019 connexin37 SNP appears to be a risk factor for ISR in the Han population of China.

Although the pathophysiological processes of CAD and ISR are different, each starts with endothelial injury induced by hemodynamic changes (shear stress) or stent placement during PCI (20). Subsequently a cascade of events occurs, including platelet and leukocyte activation which are key to atherosclerosis and SMC proliferation which is important in restenosis. Therefore, the dysfunction of the endothelial mono-layer is key to the development of CAD and ISR (21,22).

The human connexin37 gene maps to chromosome lp35.1 and encodes 333 amino acids which form the connexin37 protein. Connexin 37 is most highly expressed in the vascular endothelium, as well as in monocytes, macrophages and SMCs (23). Several connexin37 proteins form gap junction channels and hemichannels with unique properties, including distinct permeabilities for various signaling molecules (24). In an animal model, it was suggested that induced connexin37 expression may be an indicator of vascular smooth muscle cells responding to hemodynamic changes (shear stress) (25). In another animal model, connexin37^−/−^ApoE^−/−^ mice developed more aortic lesions than connexin37^+/+^ApoE^−/−^ control mice, indicating that connexin37 was atheroprotective (26). Thus, the normal connexin37 protein has a protective effect in cardiovascular diseases.

If an SNP in the connexin37 gene is a cytosine-to-thymine replacement at position 1019 (C1019T), this causes a nonconservative amino acid change in the regulatory C-terminus of the connexin37 protein, a proline-to-serine substitution (P319S) (27). This shift from proline to serine may lead to functional changes and different responses to regulatory mechanisms, such as phosphorylation. The creation of a new phosphorylation site may provide greater capacity for modulating the function from gap junctions made of this protein, which may modify endothelial cell function, and thus lead to different susceptibility to cardiovascular diseases, including CAD and ISR. However, further studies are required concerning the extensional mechanism by which allelic variants of connexin37 are differentially predictive of an increased risk of CAD and ISR.

In conclusion, the present study indicates that the C allele in the connexin37 gene may not only be associated with susceptibility to CAD, but also with restenosis following coronary stenting, particularly in the male population.

## Figures and Tables

**Table I.** Comparison of the clinical data of patients among the three groups.

Factor	Control group (n=501)	NISR group (n=465)	ISR group (n=67)
Age (years)	62.07±8.51	62.64±12.00	59.18±8.48
Gender (male/female)	334/167	376/89	56/11^a^^b^
BMI	24.48±3.25	24.53±3.36	24.49±3.41
Systolic blood pressure (mmHg)	130±21	132±18	138±25
Diastolic blood pressure (mmHg)	84±16	90±20	92±24
Fasting blood sugar (mmol/l)	5.26±0.98	5.73±1.00	5.72±0.82
Creatinine (mg/dl)	85.11±21.63	87.33±15.07	81.57±16.93
History			
Smoking (yes/no)	378/123	417/48	58/9^a^^b^
Drinking (yes/no)	346/155	356/109	49/18^a^^b^
Stent			
Diameter (mm)	-	2.98±0.67	2.79±0.87^b^
Length (mm)	-	23.46±8.59	24.16±9.23^b^
Stent release pressure (atm)	-	14.23±4.68	15.11±3.42^b^
Drug	-		
Aspirin (yes/no)	-	465/2	67/0
Clopidogrel (yes/no)	-	465/0	67/0
β-blockers (yes/no)	-	457/8	65/2
ACEI/ARB(yes/no)	-	445/20	64/3
Statins (yes/no)	-	463/2	66/1

Data expressed as mean ± SD.

a P<005 compared among the three groups.

b P<0.05 compared with the NISR group. ACEI, angiotensin-converting enzyme inhibitor; ARB, angiotensin receptor blocker; BMI, body mass index; ISR, in-stent restenosis; NISR, no in-stent restenosis.

**Table II.** Analysis of gene polymorphism test results in the CAD and control groups (%).

	Genotype				Allele	
Group	CC	CT	TT	CC+TC	C	T
CAD group (n=532)	185 (34.77)	237 (44.55)	110 (20.68)	422 (79.32)	607 (57.05)	457 (42.95)
Control group (n=501)	86 (17.16)	242 (48.30)	173 (34.53)	328 (65.47)	414 (41.32)	588 (58.68)
χ^2^			49.36	24.90		51.09
P-value			<0.01	<0.01^a^		<0.01
OR (95% CI)				2.03 (1.53–2.8)		1.89 (1.58–2.25)

Note:

a compared with TT. CAD, coronary artery disease.

**Table III.** Analysis of gene polymorphism test results in the ISR and NISR groups (%).

	Genotype				Allele	
Group	CC	CT	TT	CC+TC	C	T
ISR group (n=67)	37 (55.22)	23 (34.33)	7 (10.45)	60 (89.55)	97 (72.39)	37 (27.61)
NISR group (n=465)	148 (31.83)	214 (46.02)	103 (22.15)	362 (77.85)	510 (54.84)	420 (45.16)
χ^2^			14.89	4.89		14.72
P-value			0.001	0.027^a^		<0.01
OR (95% CI)				2.44 (1.08–5.50)		2.16 (1.45–3.22)

Note:

a compared with TT. ISR, in-stent restenosis; NISR, no in-stent restenosis.

**Table IV.** Connexin37 gene C1019T polymorphism distribution (%) in males in the CAD and control groups.

	Genotype				Allele	
Group	CC	CT	TT	CC+TC	C	T
CAD group (n=432)	140 (32.41)	204 (47.22)	88 (20.37)	344 (79.63)	484 (56.02)	380 (43.98)
Control group (n=334)	71 (21.26)	171 (51.20)	92 (27.54)	242 (72.45)	313 (46.86)	355 (53.14)
χ^2^			14.52	5.39		12.67
P-value			0.001	0.02^a^		<0.01
OR (95% CI)			-	1.48 (1.06–2.09)		1.45 (1.18–1.77)

Note:

a compared with TT. CAD, coronary artery disease.

**Table V.** Connexin37 gene C1019T polymorphism distribution (%) in females in the CAD and control groups.

	Genotype				Allele	
Group	CC	CT	TT	CC+TC	C	T
CAD group (n=100)	45 (45.00)	33 (33.00)	22 (22.00)	78 (78.00)	123 (61.50)	77 (38.50)
Control group (n=167)	15 (8.98)	71 (42.51)	81 (48.50)	86 (51.50)	101 (30.24)	233 (67.76)
χ^2^			48.95	18.54		50.20
P-value			<0.01	<0.01^a^		<0.01
OR (95% CI)				3.34 (1.90–5.86)		3.69 (2.55–5.33)

Note:

a compared with TT. CAD, coronary artery disease.

**Table VI.** Connexin37 gene C1019T polymorphism distribution (%) in males in the ISR and NISR groups.

	Genotype				Allele	
Group	CC	CT	TT	CC+TC	C	T
ISR group (n=56)	36 (64.29)	16 (28.57)	4 (7.14)	52 (92.86)	88 (78.57)	24 (21.43)
NISR group (n=376)	104 (27.66)	188 (50.00)	84 (22.34)	292 (77.66)	396 (52.66)	356 (47.34)
χ^2^			30.44	6.94		26.57
P-value			<0.01	0.008^a^		<0.01
OR (95% CI)			-	3.74 (1.32–10.64)		3.30 (2.05–5.29)

Note:

a compared with TT. ISR, in-stent restenosis; NISR, no in-stent restenosis.

**Table VII.** Connexin37 gene C1019T polymorphism distribution (%) in females in the ISR and NISR groups.

	Genotype				Allele	
Group	CC	CT	TT	CC+TC	C	T
ISR group (n=11)	1 (9.09)	7 (63.64)	3 (27.27)	8 (72.73)	9 (40.91)	13 (59.09)
NISR group (n=89)	44 (49.44)	26 (29.21)	19 (21.35)	70 (78.65)	114 (64.04)	64 (35.96)
χ^2^			7.21	0.20		4.43
P-value			0.027	0.655^a^		0.035
OR (95% CI)			-	0.72 (0.18–3.00)		0.39 (0.16–0.96)

Note:

a compared with TT. ISR, in-stent restenosis; NISR, no in-stent restenosis.
